# “Anecdotal Evidence”: Why Narratives Matter to Medical Practice

**DOI:** 10.1371/journal.pmed.0030423

**Published:** 2006-10-24

**Authors:** Rafael Campo

## Abstract

In this personal essay, Campo discusses the value of listening to patients' stories.

I want to tell you a story.

After a lecture I gave recently at a well-known medical school on the possible utility of narrative to clinical practice, from the back of the auditorium came the first question of the traditional question and answer portion of the program: “Don't you feel, Dr. Campo, that what you seem to regard as the arrogant biomedical science model of medicine is already sufficiently under attack these days?”

As the lights came up, I could make out a tall, bearded man in a long white coat, standing as if at attention near the end of one of the aisles. “We have creationists trying to teach ‘intelligent design’ in our children's science classes, and even closer to home, nurses and optometrists being given the right to prescribe medications.” Their applause having ceased, my audience now grew hushed as he went on, his voice steadily rising.

“Do you really expect physicians to accept the notion that what any ignorant patient tells us about his disease should carry a weight equal to what our years of training and expertise reveals to us about complex pathophysiology?” Then came what was clearly meant to be his coup de grace, delivered in an almost derisive tone. “Really, sir, do you have anything more than the anecdotal evidence you shared to support your thesis?”

## Our Skepticism about Anecdotes

Of course, like any physician trained in the past several decades, I too had learned to view the anecdote with the greatest amount of skepticism, if not outright disdain. The anecdote, though beguiling in its familiar engagement of our human sensibilities, is, we are all taught, the enemy of objective, dispassionate observation.

The anecdote is rife with such difficulties as openness to interpretation, and the biases of faulty memory and foolish optimism; it is just as likely to be explained by fickle chance as by anything truly under the clinician's control. It is colored by the inflections in our voices and shaped by our gestures and facial expressions. The case report counts not for academic promotion, while the randomized controlled trial of thousands of anonymous subjects has become the lingua franca of our profession, and for good reason, as rigorous epidemiologic studies have replaced mere conjecture with sound, evidence-based understanding of the causes of countless diseases and effective treatments for them. Yet to offer an anecdote these days is almost to admit the insufficiency of one's knowledge, and so we do so, at least to our fellow physicians, very apologetically.

Whether we choose to admit it or not, the anecdote continues to be an important engine of novel ideas in medicine.

## Why Narratives Matter

The inscrutably enduring power of the anecdote itself is what incites all our most fearsome defenses. So furious are we in our rejection of the merely anecdotal one cannot help but begin to wonder at it. What is it in the ostensibly harmless tale my great-grandfather told about the secret of his longevity being the small glass of bitters mixed with a raw egg he downed before bedtime each night since the age of ten that rallies us to spend billions of dollars in grants from the National Institutes of Health , disbursed every year to scientists seeking their own more explicitly pharmaceutical recipes for living longer? Why does our clinging to superstition and our willingness to be intrigued by mystery provoke such an angry, unrelenting diligence? An anecdote, after all, is just a story.

The irony in our growing intolerance of the anecdote is that storytelling is full of lessons in imagination and invention so beneficial to the creative investigator. One of my favorite, if somewhat hackneyed, anecdotes is the one told to me by my wise organic chemistry professor at Amherst College, who claimed that the discoverer of the elusive structure of benzene said his breakthrough idea of the ring of six carbon atoms linked by slithering electron bonds came to him in a wild dream he had of six snakes swallowing each others' tails. Thus we can begin to see how such dynamic constructs of narrative—the characterization of carbon atoms as lithe snakes, the metaphor of slippery bonds formed by swallowing one another's tails—animate the static concepts that perhaps frustrated more rigidly linear thinkers in their attempts to solve what had been an abiding chemical conundrum.

**Figure:**
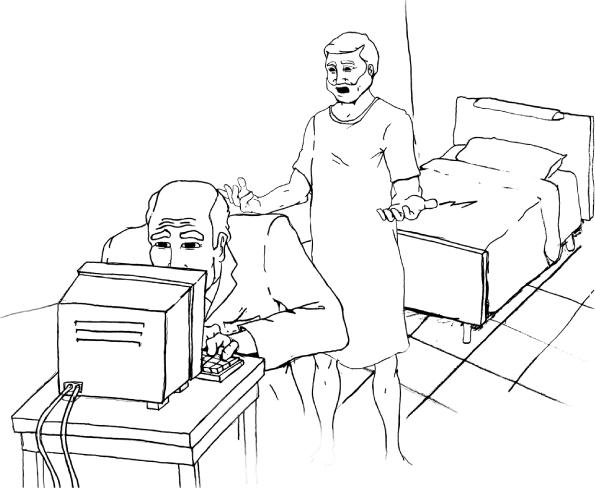
When we fail to listen to our patients' stories, we lose the opportunity to discover what truly ails them (illustration: Anthony Flores)

Whether we choose to admit it or not, the anecdote continues to be an important engine of novel ideas in medicine. No matter how wide the perceived rift between science and the humanities, and no matter what new technologies may deliver unto us in terms of more precise tests and life-prolonging therapies, the work of doctors will always necessarily take place at the intersection of science and language. How many of us have first felt inspired to dig deeper into a question that first took shape in the form of “a couple of interesting cases”—the beginnings of a case series, in epidemiological parlance—shared by a colleague over a cup of bad doctors' lounge coffee?

Our patients' stories too, if only we could listen to them less critically and cynically, might similarly inspire us to the more practically important discoveries of what truly ails them. Yes, we must always be wary of the ways in which the interlocutor may lead us astray; the possibility of violation of the narrative contract, that implicit agreement between us that the story being told is truthful and offered in the service of best care, is a real one. A patient in distress may speak to us across a chasm so vast that what we can hear is terribly distorted—by our professional distance, by our own most unprofessional fears and misapprehensions, and by society's attitudes which inescapably contextualize our every action.

One common clinical scenario has become so familiar as to be regarded as paradigmatic of our distaste for the subjective. The patient, we frequently suspect, is exaggerating her pain to obtain more narcotics, so we check to see if she is tachycardic, or whether she perspires or writhes in her sheets, ever on the lookout for more reliable objective signs of what her suspiciously anecdotal description fails to convey. Yet even in the face of language's shortcomings and betrayals, understanding narrative ultimately helps us. If we can recognize a breakdown in our communication with a suffering patient, we can begin the crucial process of repair—usually by explicitly re-establishing the ground rules of empathetic mutual trust upon which any exchange of language must be based.

Perhaps it is our own mistrust of the anecdotal that has engendered the backlash against science to which my interrogator at that recent lecture alluded. We seem to be of two minds when it comes to science as it relates to our ever defiantly human bodies. While we look to medicine to offer us the fruits of its inquiry into our innermost life-giving processes, at the same time we refuse to be entirely explicated. We want answers, but not all the answers. We want Tamiflu as well as talismans to protect us from avian influenza.

## “I Want to Tell You a Story”

The young daughter of a patient of mine wrote a poem about a flamingo, “so the birds won't get mad and make us sick.” At the bottom of the page blazed a hot pink stick figure of a bird, as if she had drawn fever itself. Might her fervent belief in the power of her own words somehow stimulate her immune system to fend off an unlucky exposure to a bird-borne virus? In all the millions of epidemiologic studies we have published in thousands of medical journals, we have yet to prove the mechanism behind a phenomenon evident in nearly all of them: the placebo effect. Perhaps there remain ideas about ourselves and our bodies that can never be summarily studied?

“I want to tell you a story,” another patient of mine said to me a few weeks later, back home in Boston, in the quieter theater of daily life. She was dying of multiple myeloma that afternoon. No more melphalan and prednisone, which had caused diabetes, nor more thalidomide, which had given her neuropathy; instead, she received only morphine now, because all that was left to treat was her pain. Rain fell relentlessly outside, streaking the windows in a way that made me think inanimate objects might somehow feel sadness. One of her daughters clutched my hand. I looked into her mother's watery, deep brown eyes, which at that moment seemed a well of stories so absorbing and so numerous that they might unspool forever. “I want to tell you a story,” she said again. Perhaps she was going to God, a notion that consoled us all; perhaps nothing was left of her but the fading impulse generated by the brain's physiology, whose final expression would be these last words. But before she could go on, her breathing stopped—leaving it all at once plainly obvious, and yet utterly incomprehensible.

